# Genetics of tibia bone properties of crossbred commercial laying hens in different housing systems

**DOI:** 10.1093/g3journal/jkac302

**Published:** 2022-12-01

**Authors:** Martin Johnsson, Helena Wall, Fernando A Lopes Pinto, Robert H Fleming, Heather A McCormack, Cristina Benavides-Reyes, Nazaret Dominguez-Gasca, Estefania Sanchez-Rodriguez, Ian C Dunn, Alejandro B Rodriguez-Navarro, Andreas Kindmark, Dirk-Jan de Koning

**Affiliations:** Department of Animal Breeding and Genetics, Swedish University of Agricultural Sciences, 756 51 Uppsala, Sweden; Department of Animal Nutrition and Management, Swedish University of Agricultural Sciences, 750 07 Uppsala, Sweden; Department of Animal Breeding and Genetics, Swedish University of Agricultural Sciences, 756 51 Uppsala, Sweden; The Roslin Institute, University of Edinburgh, Edinburgh EH25 9RG, UK; The Roslin Institute, University of Edinburgh, Edinburgh EH25 9RG, UK; Departamento de Mineralogía Y Petrologia, Universidad de Granada, 18002 Granada, Spain; Departamento de Mineralogía Y Petrologia, Universidad de Granada, 18002 Granada, Spain; Departamento de Mineralogía Y Petrologia, Universidad de Granada, 18002 Granada, Spain; The Roslin Institute, University of Edinburgh, Edinburgh EH25 9RG, UK; Departamento de Mineralogía Y Petrologia, Universidad de Granada, 18002 Granada, Spain; Department of Medical Sciences, Uppsala University, Akademiska sjukhuset, 751 85 Uppsala, Sweden; Department of Animal Breeding and Genetics, Swedish University of Agricultural Sciences, 756 51 Uppsala, Sweden

**Keywords:** bone, quantitative genetics, gene-by-environment

## Abstract

Osteoporosis and bone fractures are a severe problem for the welfare of laying hens, with genetics and environment, such as housing system, each making substantial contributions to bone strength. In this work, we performed genetic analyses of bone strength, bone mineral density, and bone composition, as well as body weight, in 860 commercial crossbred laying hens from 2 different companies, kept in either furnished cages or floor pens. We compared bone traits between housing systems and crossbreds and performed a genome-wide association study of bone properties and body weight. As expected, the 2 housing systems produced a large difference in bone strength, with layers housed in floor pens having stronger bones. These differences were accompanied by differences in bone geometry, mineralization, and chemical composition. Genome scans either combining or independently analyzing the 2 housing systems revealed no genome-wide significant loci for bone breaking strength. We detected 3 loci for body weight that were shared between the housing systems on chromosomes 4, 6, and 27 (either genome-wide significant or suggestive) and these coincide with associations for bone length. In summary, we found substantial differences in bone strength, content, and composition between hens kept in floor pens and furnished cages that could be attributed to greater physical activity in pen housing. We found little evidence for large-effect loci for bone strength in commercial crossbred hens, consistent with a highly polygenic architecture for bone strength in the production environment. The lack of consistent genetic associations between housing systems in combination with the differences in bone phenotypes could be due to gene-by-environment interactions with housing system or a lack of power to detect shared associations for bone strength.

## Introduction

Osteoporosis and bone fractures, and more generally poor bone quality, are a severe problem for the welfare of laying hens, with genetics and environment, such as housing system, each making substantial contributions to bone strength. Over their lifetimes, layers experience progressive weakening of the structural bone (Wilson *et al*. 1992; Cransberg *et al*. 2001) and increasing risk of fractures. The heritability of tibiotarsal breaking strength, one of the main phenotypes used to measure bone strength, is estimated to be around 0.2–0.5 (Bishop *et al*. 2000; González-Cerón *et al*. 2015; Mignon-Grasteau *et al*. 2016).

Housing has a fundamental and complex influence on the bones of layer hens. On the one hand, housing systems that allow for more exercise promote bone development whereas systems that restrict movement induce bone loss, as bone adapts to loading (Fleming *et al*. 1994, 2006; Newman and Leeson 1998; Leyendecker *et al*. 2005; Jendral *et al*. 2008; Shipov *et al*. 2010; Aguado *et al*. 2015; Rodriguez-Navarro *et al*. 2018). On the other hand, systems that encourage movement may also increase the fracture risk, for example, due to accidental fall from height or collision (Gregory *et al*. 1990; Abrahamsson and Tauson 1993; Fleming *et al*. 2006; Hester *et al*. 2013). Modern furnished cages allow for more movement and have a more complex environment than battery cages, but there are still environmental differences relevant to bone health between furnished cages and noncage systems (Rodenburg *et al*. 2008; Wilkins *et al*. 2011). In commercial flocks housed in aviaries with different complexity, bone strength is higher in the more complex housing systems where hens move more (Pufall *et al*. 2021). Housing system also affects the geometry, mineralization, and composition of bone, with noncaged birds having thicker and more mineralized cortical bone, and a larger amount of medullary bone, suggesting a greater capacity for bone formation in birds that can exercise more (Fleming *et al*. 2006; Shipov *et al*. 2010; Rodriguez-Navarro *et al*. 2018).

The genetic basis of bone strength in laying hens has previously been mapped in experimental intercrosses and within pedigree lines (Dunn *et al*. 2007; Raymond *et al*. 2018), but layer hens on-farm are generally crossbred and kept in different housing systems. This may make the genetic architecture of bone strength on-farm different from conditions previously studied by researchers, especially if there is gene-by-environment interaction. In particular, the genes which are involved in bone turnover in response to mechanical stimuli may differ from those involved in bone development in an environment with reduced mobility and bone loading.

In this work, we performed a genome-wide association study of tibial breaking strength, bone content and composition, as well as body weight in 860 commercial crossbred hens from 2 different companies, kept in either furnished cages or floor pens. We used a 3-point bending test, peripheral quantitative computed tomography (QCT), and thermogravimetric analysis (TGA) to estimate differences in bone strength, bone geometry, mineralization, and chemical composition between the housing systems.

## Materials and methods

### Crossbred layer hens

Crossbred layer hens of the genotypes Bovans White and Lohmann Selected Leghorn Classic (LSL) were reared at the same commercial rearing farm. Pullets destined for housing in floor pens were reared in an aviary system with full access to all tiers. Pullets destined for furnished cages were fenced in one of the tiers of the aviary to resemble rearing in a conventional rearing cage.

### Management and housing

At 15 weeks of age, the pullets were transferred to the poultry experimental facility at the Swedish Livestock Research Centre Lövsta and subsequently housed either in furnished 8-hen cages or in a 1-tier floor housing system. The housing systems and management has been described in Wall *et al*. (2022). The study was performed with ethical approval from the Uppsala Local Ethics Committee. In brief, each furnished cage provided 600 cm^2^ cage area per hen, 150 cm^2^ nest area, 150 cm litter area (on the top of the nest box), and 15 cm perch length per hen (Victorsson Industrier AB, Frillesås, Sweden). Twice a week, litter boxes were replenished with saw-dust and manure belts underneath the cage were run. Each floor pen comprised 13.4 m^2^ and was equipped with Vencomatic® one-tier system (Vencomatic Group, Eersel, The Netherlands). Two-thirds of the floor area were a raised slatted area where nests, perches, circular feed hoppers, and bell drinkers were located. The remaining floor area was covered with wood shaving. Each pen-housed 102 layers. Scrapes under the slatted area removed manure twice a week. A lighting schedule providing 9 h of light per day on arrival, with a successive increase to 14 h at 23 weeks was applied in both housing systems.

As part of the same study, we evaluated the effect of organic zinc supplementation in feed. The sampled hens were from both dietary treatments (252 treatment and 257 control in furnished cages; 224 treatment and 235 control in floor pens). As the dietary treatment was not significantly associated with bone strength (average difference of 1.7 N, *P* = 0.54 in a linear model including housing system and crossbred), we did not include diet in any of the further analyses in this paper. A detailed description of the organic zinc supplementation treatment and analyses of its effect on mortality, integument and bone strength was published in Wall *et al*. (2022).

### Bone phenotyping

At 100 weeks of age, material for bone phenotyping was collected. An intravenous injection of pentobarbital sodium (100 mg/ml) euthanized the layers. Body weight was recorded and a necropsy was conducted to make sure that only hens still in lay were chosen for bone phenotyping. The main phenotype for genome-wide association was tibiotarsal breaking strength (load to failure—we refer to it as “bone strength” for the rest of the paper).

Quantitative computerized tomography was performed with the Stratec QCT XCT Research M (Norland; v5.4B) operating at a resolution of 70 µm as previously described (Rubin *et al*. 2007). Trabecular bone mineral density, which in the female bird reflects bone mineral density of both trabecular and medullary bone, was determined ex vivo, with 2 metaphyseal QCT scans of the region situated at 6% of bone length from the distal end, and the medullary/trabecular bone was defined by setting an inner threshold to density mode (400 mg/cm^3^). In addition to medullary/trabecular bone data, scans of the metaphyseal area were also used for derivation of data for total bone. Cortical bone parameters were determined ex vivo with a mid-diaphyseal QCT scan of the tibia.

After the QCT analyses, the tibia were stored at −20°C until biomechanical testing was performed.

The tibiotarsal bones, which had previously been measured by QCT, were subsequently tested for biomechanical strength in a 3-point bending test on an electromechanical testing machine (Avalon technologies, Rochester, MN, USA). The specimens were kept frozen until a few hours prior to testing when the bones were completely thawed at room temperature. The specimens were placed with the posterior cortex resting against 2 end supports placed with a distance of 40 mm between them. The bones were placed in such a way that the load was applied 6 mm distal from the mid part of the tibiotarsal diaphysis with an anterio-posterior direction. The aim was to apply the load at the level where QCT measurements had been performed. An axial load cell (Sensotec Inc., Columbus, OH, USA) with the range 0–500 N was used to apply a load of 1 mm/s to the bone. Values for load and displacement were collected 50 times per second until failure using software provided with the testing machine (Testware II). Based on the collected data, load at failure was calculated.

Because these QCT phenotypes are highly correlated (Supplementary Fig. 1 in File 1), we used principal component analysis to reduce the QCT data to 3 principal components that we used for genome-wide association. The first principal component had high loadings for most of the radiographic phenotypes, while the second had high loadings for bone length, and the third for mostly cortical density (Supplementary Fig. 2 in File 1).

We used TGA to measure bone mineralization and composition (in cortical and medullary bone, separately), and that mainly consist of water, organic matter (collagen), and mineral (carbonate, calcium, phosphate). Powdered bones were treated at 200°C, 600°C, and 800°C in a RWF 1100 furnace (Carbolite, UK) for 1 h and weighed to determine the weight fraction of main bone chemical components. We estimated the percentage water (H_2_O%), organic matrix (organic%), mineral (mineral%) of the bone, as well as the percentage calcium phosphate (PO_4_%) and carbonate (CO_3_%) that are the main mineral part components. We calculated the degree of mineralization (PO_4_/organic) and the relative content of carbonate in the mineral (CO_3_/PO_4_). Because the thermogravimetric phenotypes are less correlated than the tomography phenotypes, we analyzed them separately instead of trying to reduce them with principal components (Supplementary Fig. 3 in File 1).

The resulting sample sizes for each set of phenotypes are shown in Supplementary Table 1.

The scanning electron microscopy images in Fig. 2 were taken from mid-diaphyseal cross-sections of the tibiae. Bones were embedded in EpoThin expoxy resin (Buehler), cut, polished, and coated with carbon (Hitachi UHS evaporator). They were imaged with FEI Quanta 400 scanning electron microscope using a backscattering electron detector.

### Genotyping

We genotyped 882 hens at 57,636 single nucleotide variants, using the Illumina Infinium assay. The genotyping was performed by the SNP&SEQ Technology Platform at Uppsala University, Uppsala, Sweden. We excluded 14 individuals with a high number of missing genotypes, as well as 19 individuals that appeared to be recorded as the wrong crossbred based on a principal component plot of the genotypes (Supplementary Fig. 4 in File 1). In order to place the SNP markers on the latest reference genome, we aligned sequences flanking the markers to the chicken reference genome version GRCg6a with BLAT (Kent 2002).

### Comparisons between housing systems

We compared bone phenotypes and body weight between housing systems using linear models including housing system and type of crossbred as covariates, and then estimated the contrast between housing systems within each crossbred. Thus, the model was:

$$y_{i}\;=\;\mu +\;\beta_{\mathrm{LSL}}x_{cb,i}+\;\beta_{\mathrm{PEN}}x_{hs,i}+\;\beta_{\mathrm{LSL}:\mathrm{PEN}}x_{cb,i}x_{hs,i}+\epsilon_{i}$$

Where *y*_*i*_ is the trait value, *μ* the coefficient for Bovans hens in furnished cages, *β*_LSL_ the coefficient for LSL hens, *β*_PEN_ the coefficient for floor pens, *β*_LSL:PEN_ coefficient for the interaction, *x*_*cb*,*i*_ and *x*_*hs*,*i*_ indicator variables for crossbreds and housing systems respectively, and *ε*_*i*_ a normally distributed error term. The contrasts of interest were −*β*_PEN_, the difference between floor pens and cages within the Bovans crossbreds, and −*β*_PEN_ − *β*_LSL:PEN_, the difference between floor pens and cages within the LSL crossbreds.

We used R statistical environment (R Core Team 2017), and the *multcomp* package for fitting linear contrasts (Hothorn *et al*. 2008).

### Genome-wide association studies

We performed genome-wide association studies using linear mixed models and a genomic relationship matrix, following the approach of Rönnegård *et al*. (2016). That is, we first used the *hglm* R package (Rönnegård *et al*. 2010) to fit a linear mixed model, and use the covariance structure for this model and ordinary least squares to fit the model for each marker efficiently.

We performed genome scans separately for each housing system and jointly, combining the housing systems. Bone phenotype scans included body mass and crossbred, and in the case of joint scans also housing system, as fixed factors. Body weight scans included crossbred, and in the joint scan also housing system, as fixed factors. Genome scans of floor pens included the pen group as a random effect. Joint scans included group as a random effect, combining all furnished cages into 1 dummy group. We used a conventional genome-wide significance threshold of 5 × 10^−8^, and a suggestive threshold of 10^−4^.

We used the same linear mixed models to estimate genomic heritability explained by the genomic relationship matrix, and perform a likelihood ratio test against a model without the additive genetic effect as a significance test of the heritability.

### Bivariate genomic models

We used GCTA to estimate genomic heritability and genomic correlations between bone breaking strength in the 2 different housing systems (Yang *et al*. 2011; Lee *et al*. 2012), analyzing the crossbreds either jointly or separately. Body weight was used as a fixed effect in all analyses, and when analyzing crossbreds together in the same model, crossbred was also included as a fixed effect. The software fits a bivariate linear mixed model using the genomic relationship matrix:

$$y_{1}=X_{1}b_{1}+\;Z_{1}g_{1}+e_{1}$$

$$y_{2}=X_{2}b_{2}+\;Z_{2}g_{2}+e_{2}$$

Where ***y***_1_ and ***y***_2_ are vectors of trait values for the two traits; ***b***_1_ and ***b***_2_ are vectors of coefficients for the fixed effects; ***g***_1_ and ***g***_2_ are vectors of additive genetic effects, ***X***_1_, ***X***_2_, ***Z***_1_, and ***Z***_2_; ***e***_1_ and ***e***_2_ are residuals. The variance–covariance matrix uses the genomic relationship matrix derived from genotypes and standardized by dividing by the global expected variance (method 1 of VanRaden 2008).

### Attempted replication of previously detected bone loci

We attempted to replicate associations from genome-wide association and linkage mapping studies of bone traits from a pedigree line and an experimental intercross (Johnsson *et al*. 2015; Raymond *et al*. 2018). The selected candidate regions are listed in Supplementary Table 2. We used genome-wide association summary statistics from markers within 50 kb of these regions.

### Overlap with previously published loci from chicken QTLdb

We used the GALLO R package (Fonseca *et al*. 2020) to perform an enrichment test with known quantitative trait loci from the Chicken QTLdb database (Hu *et al*. 2015) and a hypergeometric test. We mapped the QTL coordinates from the chicken reference genome version Galgal5.0 to GRCg6a with the UCSC LiftOver tool, which resulted in a total of 8,427 QTL that could be mapped.

## Results

### Differences between housing systems and crossbreds

**Fig. 1.**
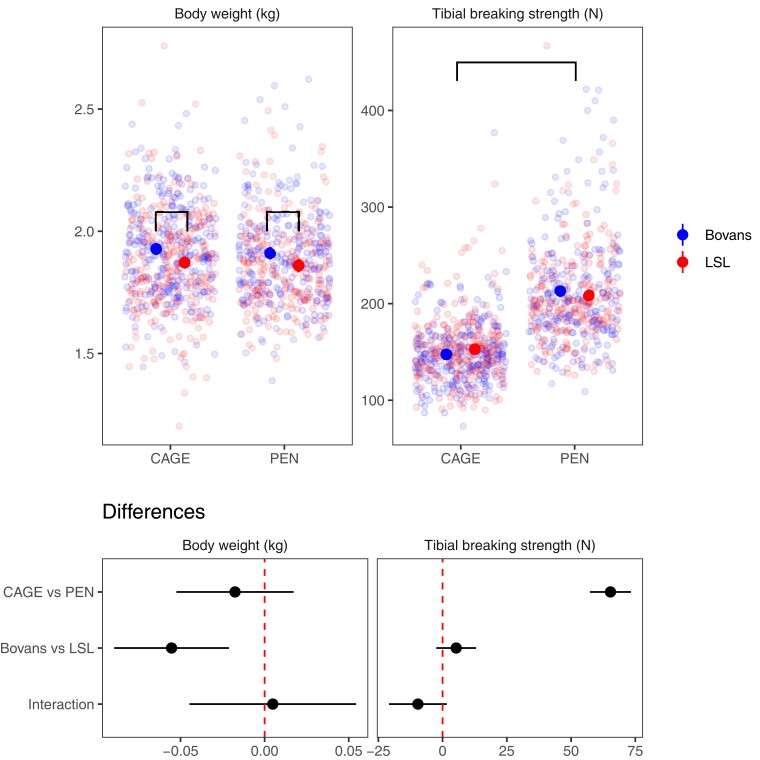
Differences in bone strength between housing systems. Body weight and tibial breaking strength broken down by housing system and crossbred, and estimates of differences between housing systems and crossbreds from a linear model including housing system, crossbred, and an interaction term. The error bars are 95% confidence intervals. The brackets indicate significant differences in body weight between crossbreds and bone breaking strength between housing systems.

**Fig. 2.**
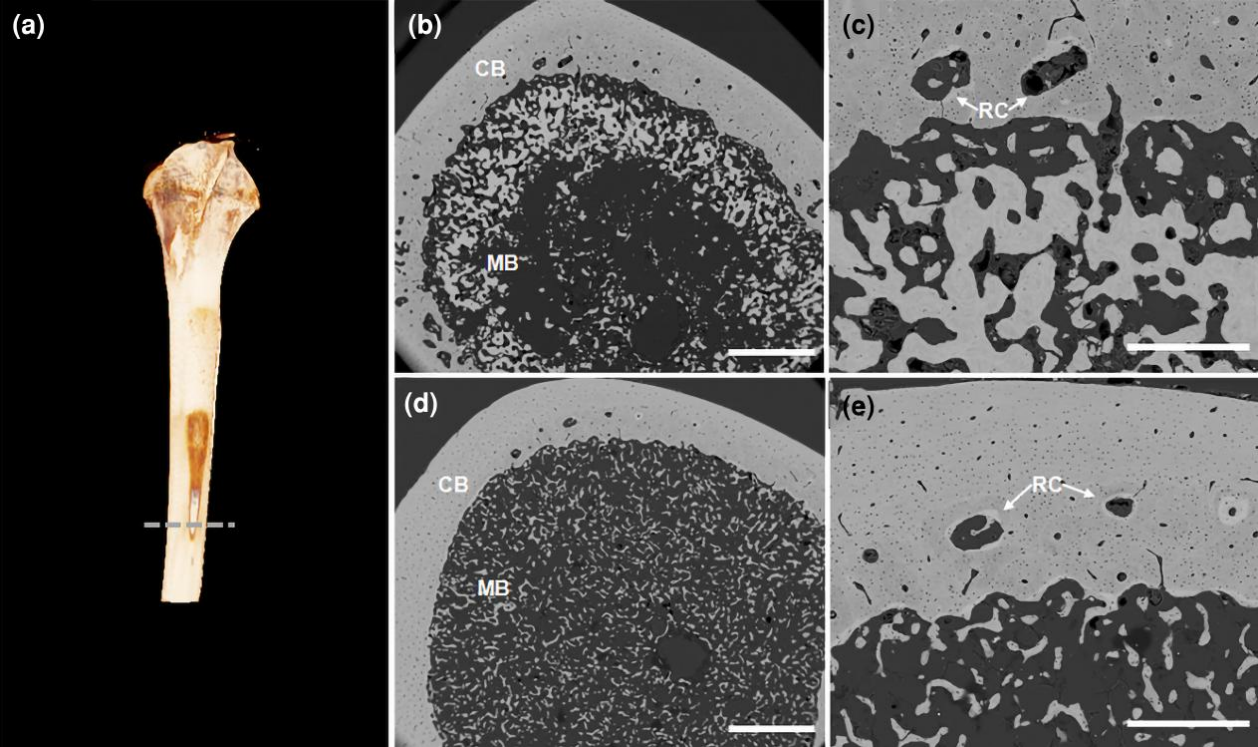
a) 3D image of a tibiae reconstructed from micro-CT. Electron backscattering images of tibia cross-section at mid-shaft from hens of different groups: PEN (b and c) and CAGE (d and e). CB, cortical bone; MB, medullary bone; RC, resorption center. Scale bar—b and d: 1 mm; c and e: 400 µm. Pen birds show a greater amount of medullary bone particles near the endosteal surface.

As expected, bone strength (load to failure) was higher (on average 65 N) in the floor pen system than in the cage system, while body weight was similar. Figure 1 shows body weight and tibial breaking strength in both housing systems and crossbreds, with estimated differences from a linear model. The crossbreds had similar tibial breaking strength, but Bovans were on average 55 g heavier than LSL hens. Figure 2 displays electron microscopy images of tibia from a hen housed in a floor pen and a hen housed in a furnished cage showing the distribution of cortical and medullary bone in cross-section. The hen housed in a floor pen had a thicker cortex and a larger amount of medullary bone than the hen from a furnished cage. Also, medullary bone particles are larger and interconnected in the floor pen whereas in furnished cage particles are smaller and isolated. These differences suggest that hens housed in floor pens have a greater capacity to form bone and mineralize the medullar cavity than hens housed in furnished cages.

**Fig. 3.**
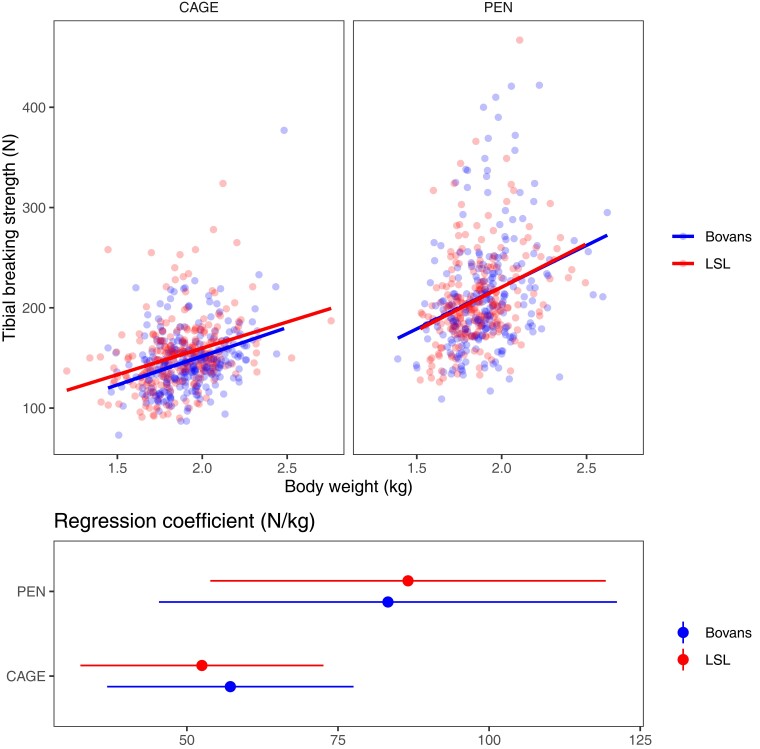
Relationship between bone strength and body weight. Tibial breaking strength and body weight broken down by housing system and crossbred and regression coefficients from a linear model within crossbred and housing system. The error bars are 95% confidence intervals.

Also as expected, there was a positive relationship between body weight and tibial breaking strength in both systems, explaining around 10% of the variance in tibial breaking strength. Figure 3 shows scatterplots of tibial breaking strength and body weight with regression coefficients from a linear model, showing a positive relationship between body weight and bone strength regardless of housing system.

**Fig. 4.**
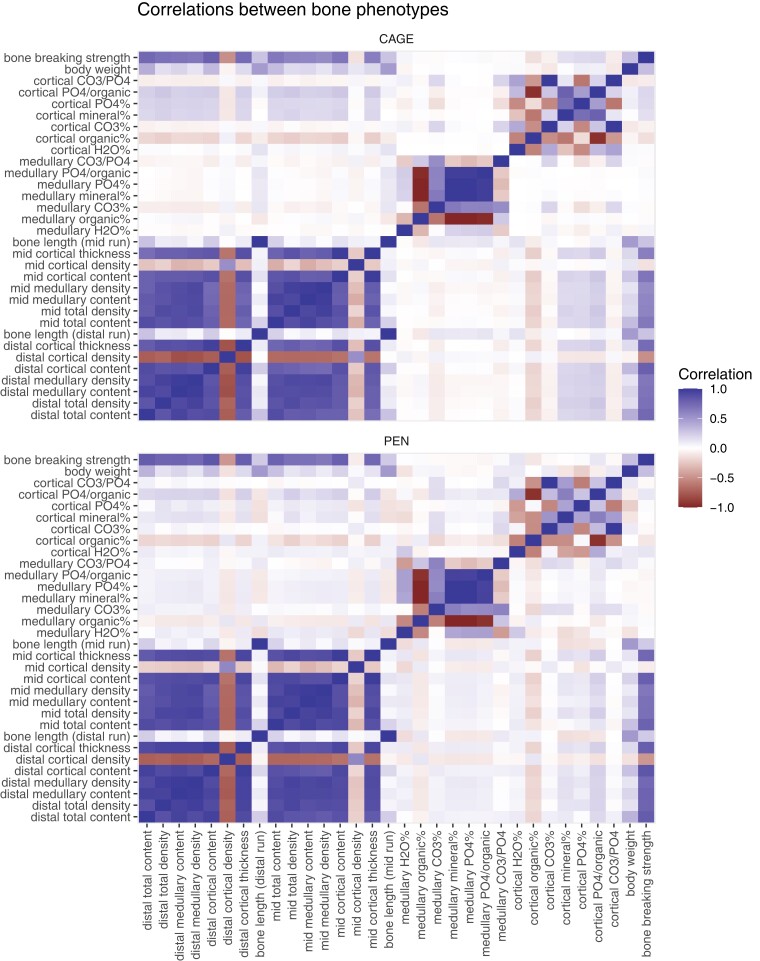
Correlations between bone phenotypes and body weight. The heatmaps show Pearson correlation of body weight, bone breaking strength, density, thickness, content, and bone composition traits, separated by housing system.

**Fig. 5.**
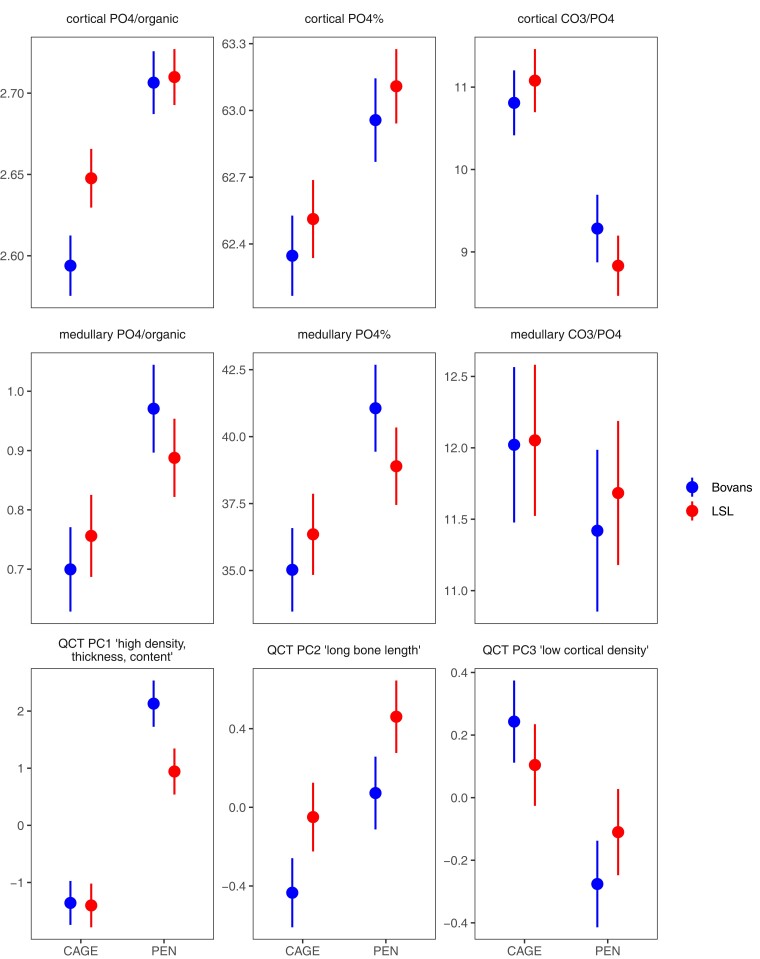
Differences in main bone phenotypes between housing systems. Estimates of means broken down by housing systems and crossbreds from a linear model including housing system, crossbred, and an interaction term, with 95% confidence intervals. All the within crossbred comparisons between housing systems, except for medullary CO_3_/PO_4_, are significant at the *P* < 0.05 level.

These differences in bone strength between housing systems were accompanied by differences in bone geometry, mineral content, cortical thickness, and bone mineral density (as measured by QCT) and chemical composition (as measured by TGA) between the housing systems. Figure 4 shows heatmaps of the correlations between these bone biomechanical properties, broken down by housing system. Figure 5 shows estimates from a linear model for the first three principal components of the QCT measurements and the main bone composition phenotypes from thermogravimetric measurements (Supplementary Fig. 4 in File 1 shows all variables). Overall, there were differences between the housing systems in most aspects of bone content and composition.

The first QCT principal component, for which cortical density, thickness and bone mineral content had the largest contributions, also show that bone quality was improved in hens housed in a floor pen. This difference was more pronounced for the Bovans crossbred than for the LSL crossbreds. Also, the tibia of hens housed in pens had cortical bone with a greater degree of mineralization and a larger amount of medullary bone than hens housed in furnished cages, as indicated by the PO_4_/organic and PO_4_% parameters determined by TGA for both types of bone (Fig. 5). Additionally, there were differences in bone chemical composition, such as the amount of carbonate (CO_3_/PO_4_) in the cortical bone mineral was significantly lower for hen housed in pens than those housed in furnished cages (Supplementary Fig. 4 in File 1).

### Heritability of bone phenotypes

Bone strength, body weight, and tomographic phenotypes had low-to-moderate genomic heritability estimates; however, most of them are not significant in a likelihood ratio test. Table 1 shows the estimated genomic heritability and the *P*-value of a likelihood ratio test for the genomic additive genetic effect.

**Table 1.** Heritability estimates for bone phenotypes and body mass, separated by housing environment and crossbred, with *p*-values from a likelihood ratio test for the genomic variance component.

		CAGE		PEN	
	Crossbred	*h* ^2^	*P*-value	*h* ^2^	*P*-value
Bone strength	Bovans	0.40	0.04	0.03	0.21
Bone strength	LSL	0.32	0.21	0.29	0.001
Body weight	Bovans	0.25	0.14	0.24	0.43
Body weight	LSL	0.24	0.17	0.23	0.27
QCT PC1 “high density, thickness, content”	Bovans	0.26	0.11	0.01	0.37
QCT PC1 “high density, thickness, content”	LSL	0.08	0.42	0.54	0.004
QCT PC2 “long bone length”	Bovans	0.06	0.37	0.09	0.43
QCT PC2 “long bone length”	LSL	0.01	0.42	0.29	0.07
QCT PC3 “low cortical density”	Bovans	0.32	0.06	0.01	0.42
QCT PC3 “low cortical density”	LSL	0.00	0.43	0.09	0.35

### Genome-wide association for bone strength and body weight

**Fig. 6.**
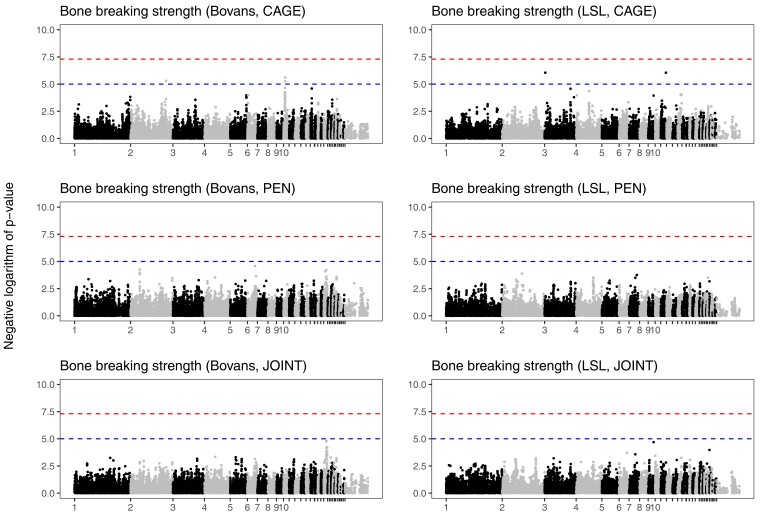
Genome-wide association of bone strength. Manhattan plots from genome scans of bone strength in each crossbred, either separating the housing systems or combining them. Bone strength scans included body mass and in the case of the joint scan also housing system as fixed effects, as well as random effects for housing groups (see Materials and methods). Chromosome names of the smaller chromosomes have been suppressed for legibility. The upper dashed line (red) shows a conventional genome-wide significance threshold of 5 × 10^−8^, and the lower dashed line (blue) a suggestive threshold of 10^−5^.

Genome scans either combining or independently analyzing the 2 housing systems detected no genome-wide significant loci for bone strength (*P* < 5 × 10^−8^), but 4 suggestive loci (*P* < 10^−5^). These loci were all detected in the furnished cage housing system only, with little evidence of a co-localizing association in the pen housing system. Two of the loci were detected only in the Bovans crossbred and the other 2 in the LSL crossbred. Figure 6 shows Manhattan plots of the genome-wide association studies for bone strength, analyzing the housing systems jointly and independently. Supplementary Figs. 6 and 7 in File 1 shows quantile–quantile plots, and Supplementary Fig. 8 a zoomed-in view of the suggestive loci.

The suggestive associations with bone strength did not overlap previously detected candidate regions for bone strength defined from other populations (Supplementary Table 2). However, there were markers with *P* < 0.01 in 3 of these regions, on chromosomes 2, 3, 8, and 23 (Supplementary Table 3).

**Fig. 7.**
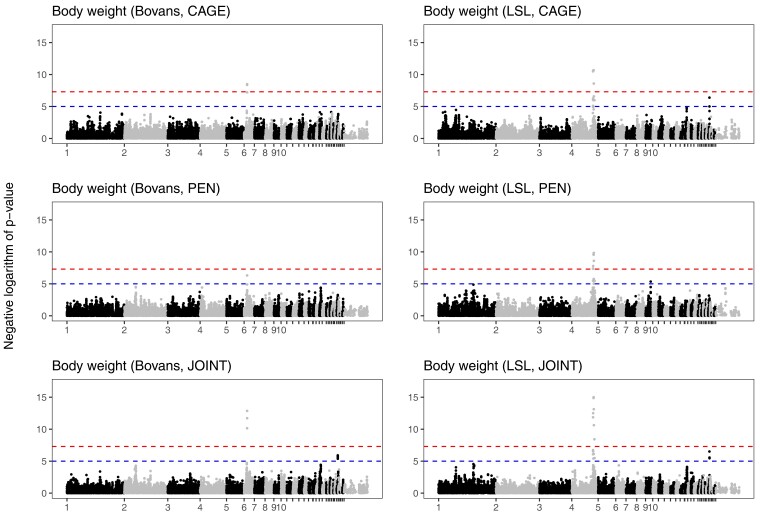
Genome-wide association of body weight. Manhattan plots from genome scans of body weight in each crossbred, either separating the housing systems or combining them. Body weight scans included crossbred and in the joint scan also housing system, as fixed covariates, as well as random effects for housing group (see Materials and methods). Chromosome names of the smaller chromosomes have been suppressed for legibility. The upper dashed line (red) shows a conventional genome-wide significance threshold of 5 × 10^−8^, and the lower dashed line (blue) a suggestive threshold of 10^−5^.

We detected 2 significant loci for body weight on chromosomes 4 and 6 as well as a suggestive locus on chromosome 27. The locus on chromosome 4 was detected only in the LSL crossbred, while the locus on chromosome 6 was detected only in the Bovans crossbred, and the locus on chromosome 27 was suggestive in both crossbreds. Supplementary Fig. 9 in File 1 shows a zoomed-in view of the 3 body weight loci (Fig. 7). Table 2 shows the locations of associations. Because the chromosome 4 locus contains multiple significant markers spread over a region of several megabasepairs, we performed a conditional scan that included the most significant marker in the region as a covariate (Supplementary Fig. 10 in File 1). Controlling for the most significant marker abolished the significant association throughout the whole region, meaning that we have no clear evidence of multiple linked loci in the region.

**Table 2.** Overview of significant and suggestive (*p* < 10^−5^) regions from genome-wide association scans.

Trait	Chromosome	Lead SNP position	Lead SNP *P*-value
*Significant*			
Body weight (LSL, CAGE)	4	75151189	2.14E-11
QCT PC2 “long bone length” (LSL, CAGE)	4	75151189	1.74E-10
Body weight (LSL, JOINT)	4	75748329	9.99E-16
QCT PC2 “long bone length” (LSL, JOINT)	4	75748329	3.58E-13
Body weight (LSL, PEN)	4	76229679	1.45E-10
QCT PC3 “low cortical density” (LSL, CAGE)	4	76229679	1.95E-10
Body weight (Bovans, CAGE)	6	11477631	3.19E-09
Body weight (Bovans, JOINT)	6	11477631	1.38E-13
QCT PC2 “long bone length” (Bovans, CAGE)	6	11477631	6.63E-12
QCT PC2 “long bone length” (Bovans, JOINT)	6	11477631	2.00E-14
QCT PC2 “long bone length” (LSL, JOINT)	27	6070932	2.63E-08
*Suggestive*			
QCT PC3 “low cortical density” (LSL, CAGE)	1	123408718	3.52E-06
QCT PC1 “high density, thickness, content” (LSL, CAGE)	1	131958610	1.18E-06
QCT PC2 “long bone length” (Bovans, JOINT)	2	55376377	1.12E-07
QCT PC2 “long bone length” (Bovans, PEN)	2	55376377	5.45E-07
Bone strength (Bovans, CAGE)	2	125198709	4.93E-06
Bone strength (LSL, CAGE)	3	2430687	8.81E-07
QCT PC3 “low cortical density” (LSL, JOINT)	3	50377056	2.85E-06
QCT PC3 “low cortical density” (Bovans, JOINT)	3	88093788	4.10E-06
QCT PC2 “long bone length” (LSL, PEN)	4	75748329	4.60E-07
QCT PC3 “low cortical density” (LSL, JOINT)	4	76229679	1.13E-06
QCT PC1 “high density, thickness, content” (Bovans, JOINT)	5	40497275	5.94E-06
Body weight (Bovans, PEN)	6	11477631	4.86E-07
QCT PC2 “long bone length” (Bovans, PEN)	6	11477631	4.20E-06
QCT PC1 “high density, thickness, content” (LSL, CAGE)	6	33580943	3.93E-06
QCT PC3 “low cortical density” (Bovans, CAGE)	9	8939638	4.64E-06
Body weight (LSL, PEN)	9	18273076	4.47E-06
Bone strength (Bovans, CAGE)	10	7029216	2.53E-06
Bone strength (LSL, CAGE)	11	18432763	8.81E-07
QCT PC1 “high density, thickness, content” (LSL, JOINT)	19	7292331	5.42E-06
QCT PC1 “high density, thickness, content” (LSL, CAGE)	26	2251364	9.40E-07
Body weight (LSL, CAGE)	27	6070932	4.10E-07
Body weight (LSL, JOINT)	27	6070932	3.09E-07
QCT PC2 “long bone length” (LSL, PEN)	27	6070932	1.78E-06
Body weight (Bovans, JOINT)	27	6087051	1.24E-06

**Fig. 8.**
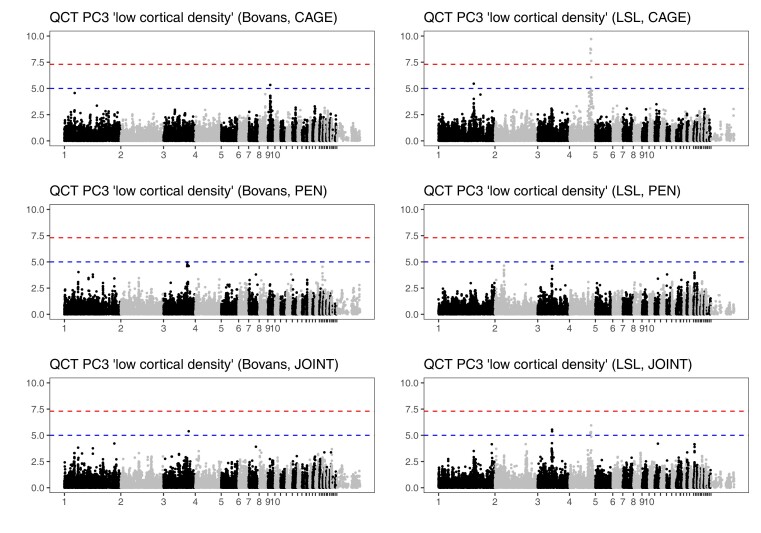
Genome-wide association of third principal components of QCT phenotypes. Genome scans included body mass and in the case of the joint scan also housing system as fixed effects, as well as random effects for housing groups (see Materials and methods). Chromosome names of the smaller chromosomes have been suppressed for legibility. The upper dashed line (red) shows a conventional genome-wide significance threshold of 5 × 10^−8^, and the lower dashed line (blue) a suggestive threshold of 10^−5^.

Genome scans for the QCT phenotypes detected 6 significant and 15 suggestive associations. Figure 8 shows Manhattan plots of the third principal component, and Supplementary Figs. 11 and 12 in File 1 show Manhattan plots of the remaining 2. Table 2 and Supplementary Table 5 summarize the location of significant and suggestive regions, respectively. The significant associations with principal components 2 and 3 coincide with body weight loci on chromosomes 4, 6, and 27.

### Genetic differences between housing systems

**Fig. 9.**
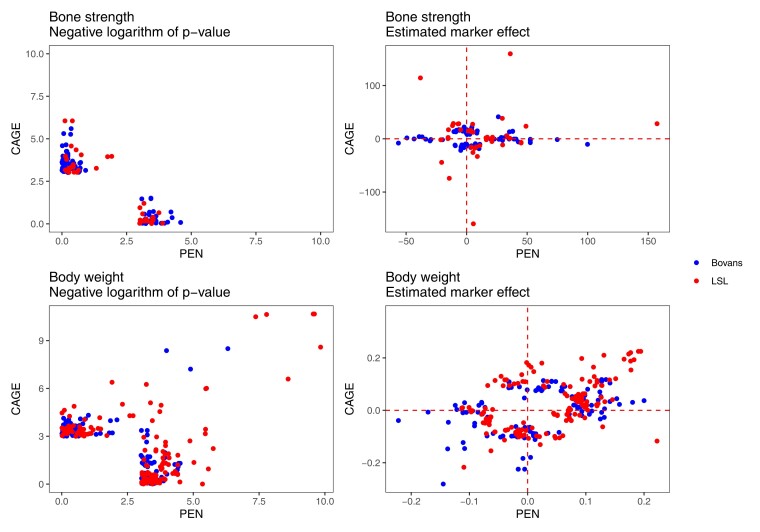
Comparison of genetic associations between housing systems. Scatterplots compare the *P*-values and estimated marker effects of markers with *P* < 10^−3^ either in furnished cages or in floor pens.

There was no overlap between the suggestive loci for bone strength in the 2 housing systems. Figure 9 compares the *P*-values and estimated marker effects, using all markers with *P* < 10^−3^ between the floor pen and furnished cage systems. For comparison, we also show the same scatterplots for the body weight scan, where the loci overlap between housing systems.

Genetic correlation estimates between housing systems were too imprecise to be useful. We used a bivariate model with the genomic relationship matrix to estimate genomic heritability and correlation between housing systems. Supplementary Table 4 shows the estimated genetic correlations and heritabilities from this model.

## Discussion

In this paper, we found that bone strength in commercial crossbred laying hens is highly polygenic and potentially exhibits gene-by-environment interactions between housing systems that allow different amounts of exercise. We detected no genome-wide significant loci for bone strength, and the suggestive loci were different between the 2 environments. In contrast, we detected 3 significant body weight loci shared between environments and coincided with significant loci for bone length. This leads to 3 topics for discussion:

1. differences in bone strength, content, and composition between floor pen and furnished cage housing systems;
2. evidence for gene-by-environment interaction between housing systems; and
3. candidate genes underlying loci for body weight and bone length.

### The effect of housing system on bone strength, content, and composition

Our detailed bone phenotyping revealed several differences in bone strength, content, and chemical composition between hens housed in furnished cages and hens housed in floor pens. The environmental difference between housing systems causes a quantitative increase in bone strength accompanied by increased bone formation and mineralization in the floor pen system, where the hens are able to exercise more.

In addition to greater bone strength, the QCT results show the principal component containing predominately cortical density, cortical thickness, and bone mineral content being improved in a pen environment. Previous results also demonstrated increased bone cortical thickness, a lower bone cortical porosity, a larger amount of medullary bone, and overall a greater total bone mass as factors contributing to the greater strength seen in hens housed in aviary systems that also allowed for greater mobility (Fleming *et al*. 2006; Shipov *et al*. 2010; Rodriguez-Navarro *et al*. 2018). Also, analysis by thermogravimetry shows that hens housed in floor pens have a higher degree of bone mineralization. The main traits describing the amount of bone mineralization of cortical and medullary bone (PO_4_/organic and PO_4_%) were greater in hens housed in floor pens than in furnished cages. This is consistent with previous results, as the greater opportunity for physical exercise stimulates bone formation and increases mineralization of the medullary cavity (Shipov *et al*. 2010; Rodriguez-Navarro *et al*. 2018).

On the other hand, hens in floor pens had bone with a greater degree of mineralization and a higher carbonate/phosphate ratio than hens housed in cages. A greater degree of mineralization and lower carbonate/phosphate ratio is indicative of an increased bone maturity and lower turnover rates reflecting a decreased amount of remodeling of established bone in hens in floor pens. In contrast, Rodriguez-Navarro *et al*. (2018) found that hens with increased mobility had cortical bone with lower degree of mineralization and higher carbonate/phosphate ratio, suggesting a higher amount of bone remodeling. Thus, it seems the effect of exercise on bone remodeling and maturation also depends on other factors, such as age or other environmental variables.

This discrepancy in the response of bone to physical activity might be explained by aging effects, if a higher metabolic activity in pen-housed chickens at an earlier age coincides with, or even causes, a lower metabolic activity at a later age. The hens in Rodriguez-Navarro *et al*. (2018) were 56 weeks old at sampling and should have a more active bone metabolism than hens in this study that were 100 weeks old. The differences in bone strength and geometry might have been established at an earlier age. Bone metabolism is a dynamic process where what happened earlier in life matters. For example, bone quality is negatively genetically correlated with age at first egg, suggesting that early sexual maturation causes worse bone quality later in life (Dunn *et al*. 2021). Similarly, whether pullets are reared in cages or in aviaries, allowing for more movement, has long-term effects on bone properties later in life (Casey-Trott *et al*. 2017). This suggests that longitudinal studies of bone mineralization and remodeling in layer hens are warranted.

As we have observed before, the medullary bone shows more pronounced effects than cortical bone. Thus, it appears that medullary bone responds to exercise even at older age, despite contributing less to bone strength than cortical bone. This is in accordance with previous results: Medullary bone composition had significant heritabilities in white and brown egg layers (Dunn *et al*. 2021), and medullary bone has showed increased PO_4_/amide levels in response to exercise (Shipov *et al*. 2010; Rodriguez-Navarro *et al*. 2018). Previous studies also suggested that the amount of medullary bone was increased by the selection for better bone quality and by increased physical activity in aviary systems (Fleming *et al*. 2006). Medullary bone was clearly more mineralized in both crossbreds when housed in pens. In this study, there is little apparent correlation between medullary bone and bone strength, but other studies have found association between medullary mineralization and bone strength (Rodriguez-Navarro *et al*. 2018; Alfonso-Carrillo *et al*. 2021; Dunn *et al*. 2021). Thus, variation in medullary bone is an important contributor to variability in bone mineral content and mechanical properties, both in terms of genetic variation and response to exercise.

### The evidence for gene-by-environment interaction between housing systems

Genome-wide association scans of bone strength gave completely different results between hens housed in furnished cages and hens housed in floor pens, suggesting that the genetic basis of bone strength may be different in the 2 housing systems. There were no suggestive associations in common between the 2 housing systems, and little concordance between estimated marker effects. In combination with evidence for differences in bone content and composition between housing systems, we hypothesize that this difference may be due to gene-by-environment interaction. That is, the genetic architectures of bone strength in a furnished cage and in a floor pen could be different, likely because these environments put such different pressures on bone development and homeostasis. Therefore, the genes involved in bone turnover in response to loading may be substantially different to those involved in contributing to variance where loading is less.

On the contrary, the genome-wide association results for body weight were consistent between the housing systems. This similarity suggests that the genetic variants that affect growth, at least at the three loci detected in this study, do not interact with the housing system. At the same time, there was little difference in average body weight between hens in the 2 housing systems.

However, low power to detect associations for bone strength is also a possible alternative explanation for this pattern of an absence of shared associations. It is possible that there are loci that are shared between housing systems but that have too small effects to be reliably detected in both genome scans. Similarly, the point estimates of the genetic correlations between housing systems, both within crossbred and combined, were high but their standard errors are large enough to be consistent with both high and low correlations. Thus, it seems that larger sample sizes are needed to address the question about gene-by-environment interaction between housing systems.

### Candidate genes for body weight and bone length

The body weight loci on chromosomes 4, 6, and 27 overlap loci reported in several previous genetic mapping studies. The regions overlap several compelling candidate genes for body weight in chickens, which is also reflected in enrichment of body weight and feed conversion associations from Chicken QTLdb (Supplementary Fig. 13 in File 1). This includes studies within laying hen populations where the same region on chromosome 4 was seen to also have pleiotropic effects on a wide range of traits including egg quality traits (Wolc *et al*. 2014).

Two different loci for body weight overlapping our chromosome 4 locus have been fine mapped down to regions of one or a few candidate genes. A series of genetic mapping studies (Nassar *et al*. 2015; Lyu *et al*. 2017, 2018) detected and progressively fine mapped a region containing 15 genes, including *Ligand-dependent nuclear receptor corepressor like* (*LCORL; ENSGALG00000014421*) and *Condensin complex subunit 3* (*NCAPG*; *ENSGALG00000014425*). This locus is also associated with body size traits in humans (Weedon *et al*. 2008), cattle (Bouwman *et al*. 2018), and horses (Makvandi-Nejad *et al*. 2012). The other locus was detected by Sewalem *et al*. (2002) and fine mapped to *Cholecystokinin receptor type A* (*CCKAR*; *ENSGALG00000030801*), and was shown to alter the expression of the *CCKAR* gene and the physiological response of the animals to its ligand CCK (Dunn *et al*. 2013). The associated region found in this study overlaps both of these regions. One or both of them might contribute to the association; due to linkage disequilibrium, we cannot tell them apart. We confirmed this by a conditional genome-wide association scan, where adding the lead SNP as a covariate abolished the association signal throughout the region. This suggests that linkage disequilibrium throughout the region prevents us from genetically dissecting it further in this population. This region appears to be a hotspot of genetic effects on body weight, or perhaps more correctly stature, across a large range of animals, with pleiotropic effects on other traits.

The 2 most significant associations on chromosome 27 fall in the *insulin-like growth factor 2 mRNA binding protein 1* gene (*IGF2BP1*; *ENSGALG00000041204*). *IGF2BP1* is known to be expressed in developing limbs and has been shown to alter the length of chick long bones (Fisher *et al*. 2005) which could ultimately affect stature. The *IGF2BP1* locus has been highlighted previously in a GWAS study in a population of laying type chicken which included white leghorns genetics and affected a range of carcase traits including feet weight with effects up to 4.78% of the variance (Ma *et al*. 2019). The study also demonstrated the region between *CCKAR* and *NACPG* as important for carcase traits as in this study. Expression of *IGF2BP1* is also associated with adipogenesis in chickens (Chen *et al*. 2019). This association is also close to bone candidate gene *sclerotin* (*SOST; ENSGALG00000009929*), located about 150 kb way. Sclerotin is a negative regulator of bone formation that is expressed in osteocytes (van Bezooijen *et al*. 2005); loss-of-function mutations in humans cause bone overgrowth (sclerosteosis). Guo *et al*. (2017) report an association with femoral bone mineral content and femoral weight in this region, highlighting *SOST* as a candidate gene. For femoral weight on their lead SNP occurs close to *IGF2BP1*, while their lead SNP for bone mineral content is closest to *SOST*.

We detected significant loci associated with bone length coinciding with the major body weight loci, despite including body weight as a covariate in the bone length genome scan. This may be an artefact of a nonlinear relationship between body weight and bone length, or a genuinely pleiotropic effect on bone length. However, there was one association for bone length independent of body weight on chromosome 2. The closest gene was *succinyl-CoA:glutarate-CoA transferase* (*SUGCT; ENSGALG00000031758*). This gene encodes a mitochondrial enzyme that is associated with glutaric aciduria in humans, but appears to have no known connection to bone or to body size traits.

### Conclusion

The current study yet again establishes the positive effects of systems that allow greater movement of laying hens on bone quality, and that these beneficial effects can also be seen in old hens (100 weeks of age). Knowledge acquired in this study could help in moving to selection strategies aimed to reduce the incidence of bone damage in laying hens in systems that allow greater mobility. This could allow phenotypes gathered in extensively housed hens be applied to pedigree hens, which may need to be selected in a cage environment for egg laying performance. This could conceivably be achieved by genomic selection or by sib selection.

## Supplementary Material

jkac302_Supplementary_Data

## Data Availability

The underlying data have been deposited to figshare with doi 10.6084/m9.figshare.14405894 (http://dx.doi.org/10.6084/m9.figshare.14405894), containing 1 file of SNP chip genotypes; 1 phenotype file of bone traits, body weight, and covariates; a file mapping phenotype column names to the trait names used in the article; and 1 file of marker positions. The summary statistics of all genome-wide association studies have been deposited to Figshare with doi 10.6084/m9.figshare.21340734 (https://dx.doi.org/10.6084/m9.figshare.21340734). The analysis scripts are available at https://github.com/mrtnj/layer_bone_gwas. Supplemental material available at *G3* online.

## Notes

*Communicating editor: G. de los Campos*
