# Supplementary material for: Genetics of tibia bone properties of crossbred commercial laying hens in different housing systems
Source: G3 (Bethesda). 2022 Dec 1;13(2):jkac302. doi: 10.1093/g3journal/jkac302 (PMC9911068; doi:10.1093/g3journal/jkac302)
Supplement: jkac302_Supplementary_Data [file jkac302_supplementary_data.zip › File_S1_G3-2022-403923.docx]

# Supplementary figures

Figure S1. Correlation heatmap and variance explained by principal components of QCT phentoypes.

Figure S2. Loadings on the first three principal components of the QCT phenotypes, showing how the first captures most density and content variables, the second tibial bone length, and the third cortical density.

Figure S3. Correlation heatmap and variance explained by principal components of TGA phentoypes.

Figure S4. Differences in bone phenotypes between housing systems. Estimates of differences between housing systems and crossbreds from a linear model including housing system, crossbred and an interaction term. Differences are expressed a linear contrast between housing systems (cage minus pen) within the two crossbreds (LSL and Bovans). Thus, positive values mean that trait values are higher, on average, in furnished cages than in floor pens, and vice versa. The red dashed line indicates zero; intervals that do not overlap this line are significantly different from zero.

Figure S5. The first principal component separates the two crossbreds. Scatterplot of the first two principal components of the genotypes, coloured by the crossbred. 19 individuals appeared to be recorded as the wrong crossbred based on the position the plot, and were excluded.


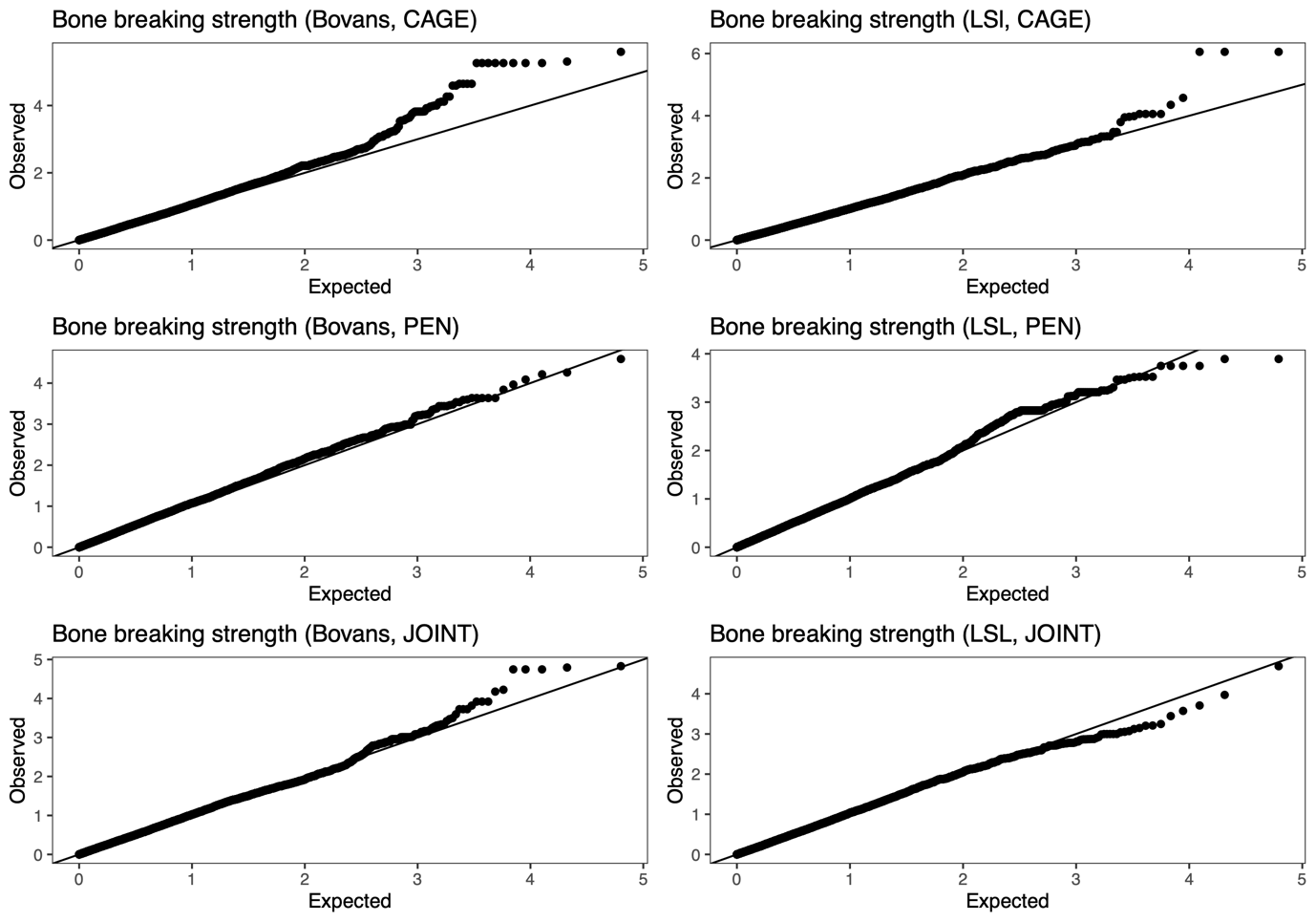


Figure S6. Quantile—quantile plots of genome scans for bone breaking strength.


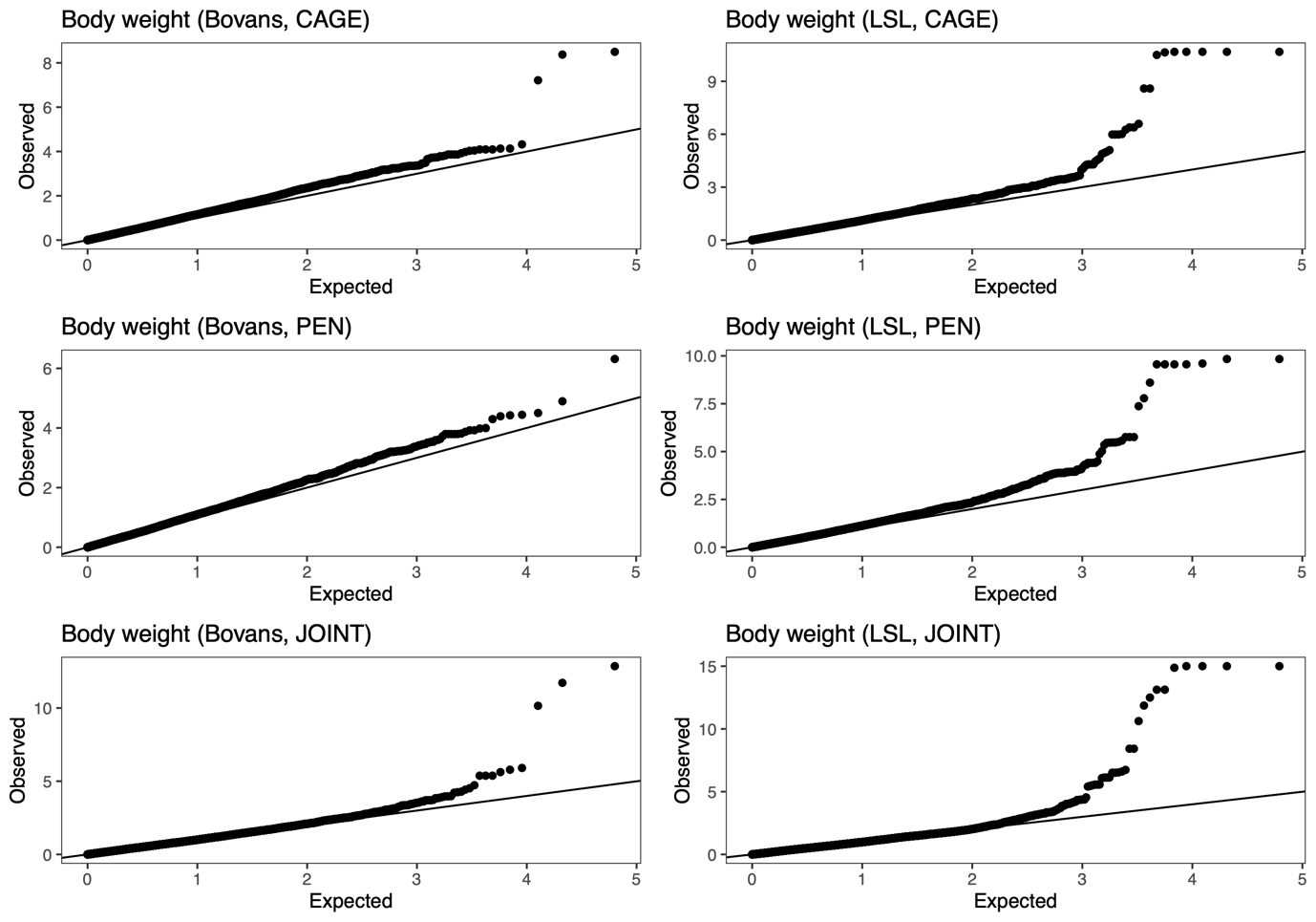


Figure S7. Quantile—quantile plots of genome scans for body weight.

Figure S8. Zoomed-in view of suggestive genome-wide associations for bone breaking strength. The dashed blue line shows a suggestive threshold of 10^-5^.

Figure S9. Zoomed-in view on genome-wide associations for body weight. The dashed blue line shows a conventional genome-wide significance threshold of 5 * 10^-8^.

Figure S10. Conditional GWAS of the chromosome 4 locus for body weight in the LSL crossbred. The plot shows the negative logarithm of the p-value for chromosome 4, with black dots being the joint GWAS performed in the main analysis, and grey dots a conditional GWAS including the lead SNP from the locus. This conditional scan removes associations throughout the region.


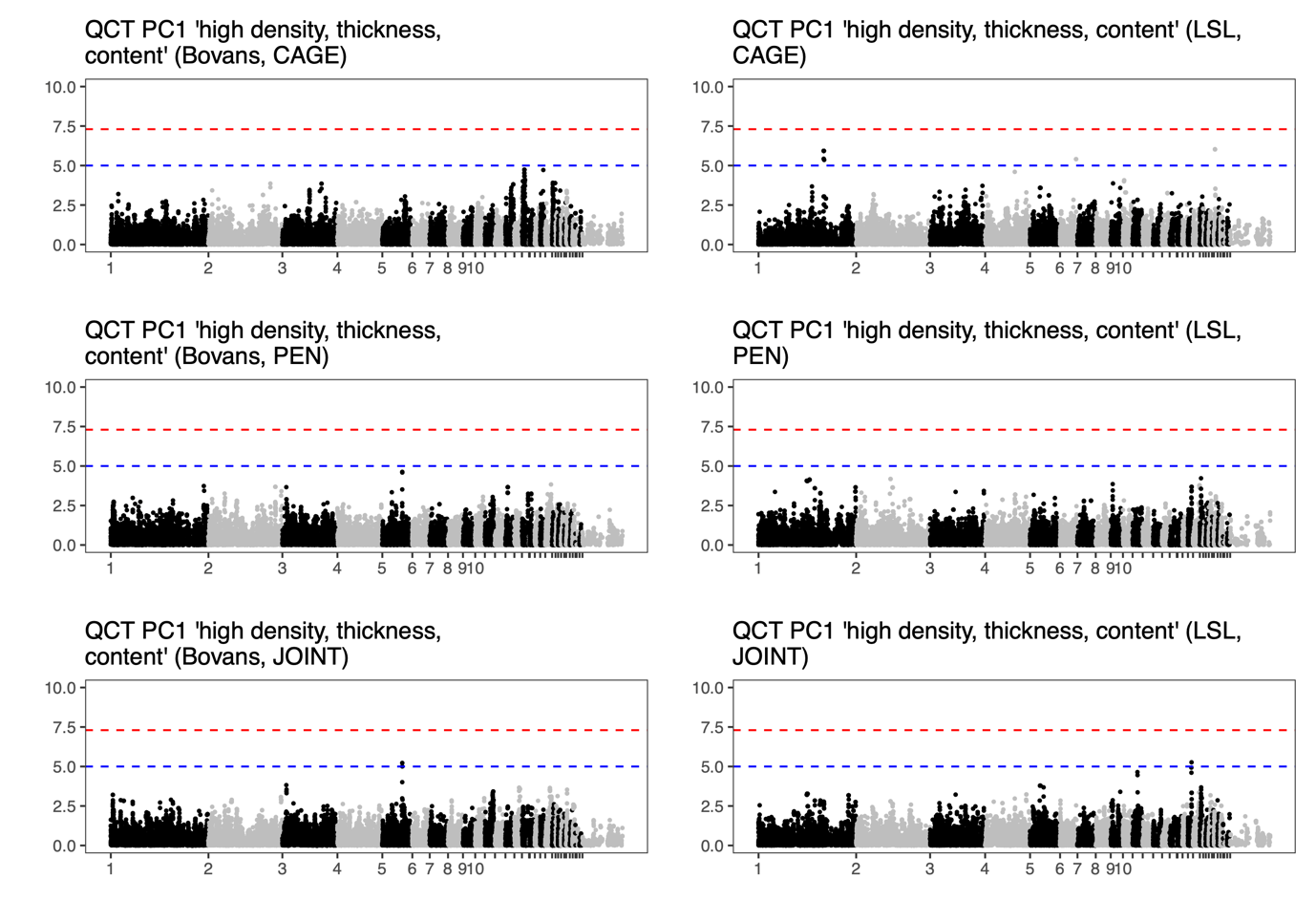


Figure S11. Genome-wide association of first principal components of QCT phenotypes. Genome scans included body mass and in the case of the joint scan also housing system as fixed effects, as well as random effects for housing groups (see Methods). Chromosome names of the smaller chromosomes have been suppressed for legibility. The dashed red line shows a conventional genome-wide significance threshold of 5 * 10^-8^, and the dashed blue line a suggestive threshold of 10^-5^.


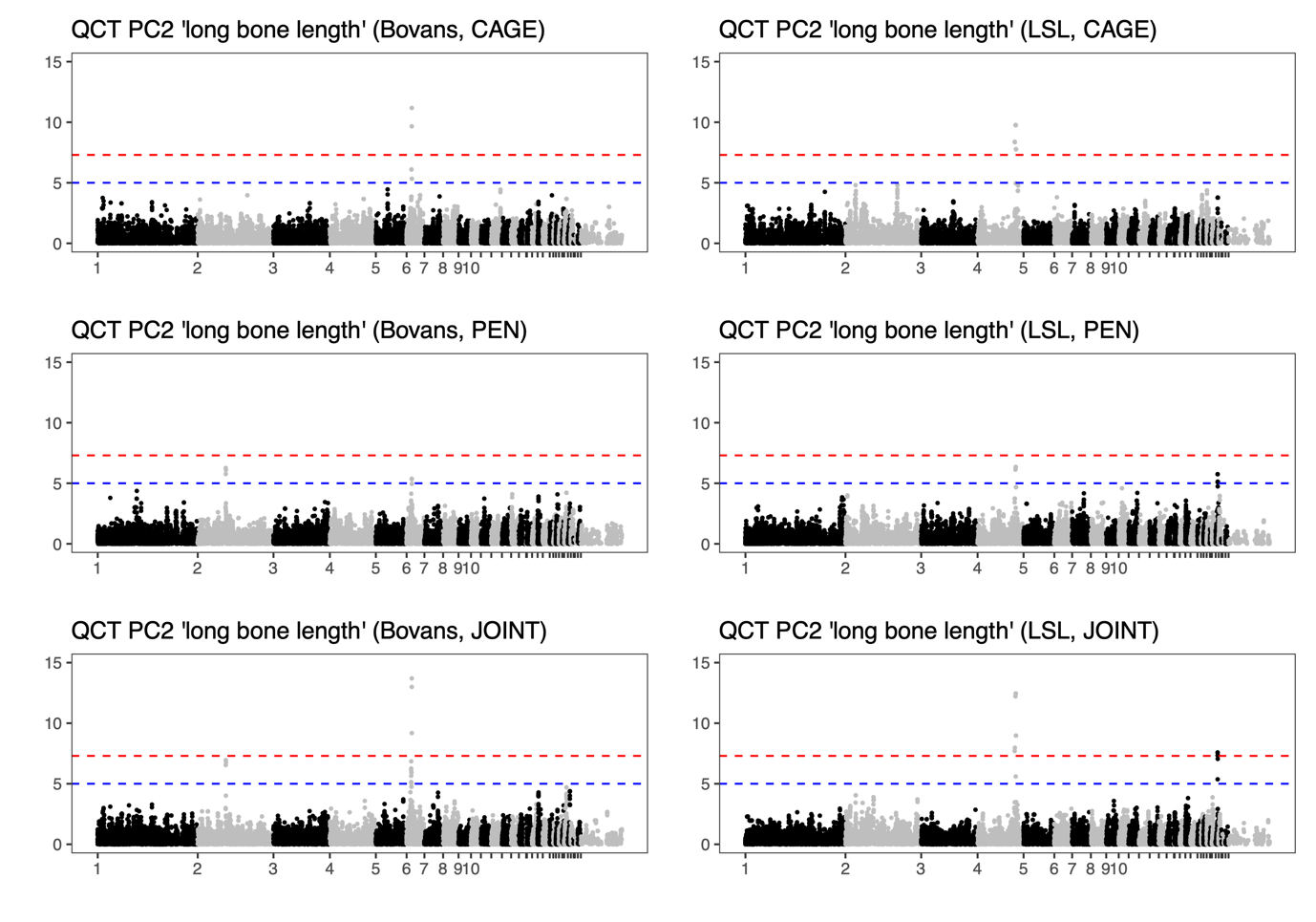


Figure S12. Genome-wide association of second principal components of QCT phenotypes. Genome scans included body mass and in the case of the joint scan also housing system as fixed effects, as well as random effects for housing groups (see Methods). Chromosome names of the smaller chromosomes have been suppressed for legibility. The dashed red line shows a conventional genome-wide significance threshold of 5 * 10^-8^, and the dashed blue line a suggestive threshold of 10^-5^.

Figure S13. Enrichment of previously published QTL from the Chicken QTLdb database overlapping significant body weight and suggestive bone strength associations.
