# Supplementary material for: Genetics of tibia bone properties of crossbred commercial laying hens in different housing systems
Source: G3 (Bethesda). 2022 Dec 1;13(2):jkac302. doi: 10.1093/g3journal/jkac302 (PMC9911068; doi:10.1093/g3journal/jkac302)
Supplement: jkac302_Supplementary_Data [file jkac302_supplementary_data.zip › Supplemental_Table_Legends_G3-2022-403923.docx]

# Description of supplementary tables

Supplementary Table S1. Sample sizes.

Supplementary Table S2. Predefined candidate regions derived from previous studies.

Supplementary Table S3. Markers in predefined candidate regions with p < 0.01.

Supplementary Table S4. Genomic heritabilities and correlations from bivariate model.

Supplementary Table S5. Suggestive associations from genome-wide association studies.
